# H_2_S/Butane Dual Gas Sensing Based on a Hydrothermally Synthesized MXene Ti_3_C_2_T_x_/NiCo_2_O_4_ Nanocomposite

**DOI:** 10.3390/molecules29010202

**Published:** 2023-12-29

**Authors:** Shama Sadaf, Hongpeng Zhang, Ali Akhtar

**Affiliations:** 1 Marine Engineering College, Dalian Maritime University, Dalian 116026, China; shamasadaf10@gmail.com; 2 Hangzhou Institute of Advanced Studies, Zhejiang Normal University, Hangzhou 311231, China; aliakhtar6091995@gmail.com; 3 Zhejiang Institute of Photo-Electronics, Zhejiang Normal University, Jinhua 321004, China

**Keywords:** MXene Ti_3_C_2_T_x_, spherical NiCo_2_O_4_, sensor sheets, butane sensing, ppb-level H_2_S-sensing

## Abstract

Real-time sensing of hydrogen sulfide (H_2_S) at room temperature is important to ensure the safety of humans and the environment. Four kinds of different nanocomposites, such as MXene Ti_3_C_2_T_x_, Ti_3_AlC_2_, WS_2_, and MoSe_2_/NiCo_2_O_4_, were synthesized using the hydrothermal method in this paper. Initially, the intrinsic properties of the synthesized nanocomposites were studied using different techniques. P-type butane and H_2_S-sensing behaviors of nanocomposites were performed and analyzed deeply. Four sensor sheets were fabricated using a spin-coating method. The gas sensor was distinctly part of the chemiresistor class. The MXene Ti_3_C_2_T_x_/NiCo_2_O_4_-based gas sensor detected the highest response (16) toward 10 ppm H_2_S at room temperature. In comparison, the sensor detected the highest response (9.8) toward 4000 ppm butane at 90 °C compared with the other three fabricated sensors (Ti_3_AlC_2_, WS_2_, and MoSe_2_/NiCo_2_O_4_). The MXene Ti_3_C_2_T_x_/NiCo_2_O_4_ sensor showed excellent responses, minimum limits of detection (0.1 ppm H_2_S and 5 ppm butane), long-term stability, and good reproducibility compared with the other fabricated sensors. The highest sensing properties toward H_2_S and butane were accredited to p–p heterojunctions, higher BET surface areas, increased oxygen species, etc. These simply synthesized nanocomposites and fabricated sensors present a novel method for tracing H_2_S and butane at the lowest concentration to prevent different gas-exposure-related diseases.

## 1. Introduction

Developing novel gas sensors for detecting hazardous gases (H_2_S, C_2_H_4_, butane, NO_2_, SO_2_, and NH_3_) is essential due to their variety of applications for human safety and the ecological environment. Hydrogen sulfide (H_2_S), with an unpleasant pungent smell, is one of the most nocuous air pollutants. It is also called industrial exhaust gas, which can be released from eggs, meat, wastewater treatment centers, sewers, and oil and gas fields [1,2]. The Occupational Safety and Health Administration (OSHA) regulation limits the allowed concentration of 100 ppb H_2_S in air [3]. Different concentrations of H_2_S exposure such as 2–5 ppm, 100–150 ppm, 200–300 ppm, 500–700 ppm, and 700–2000 ppm cause headaches, nausea, loss of smell, pulmonary edema, loss of consciousness, and sudden death within a minute, respectively [4]. That is why it is very important to establish real-time H_2_S detection. On the other hand, butane (C_4_H_10_) is a colorless and flammable gas that normally exists in wet/cracking gas, which could be considered one of the primary components of liquefied petroleum gas (LPG). Similar to H_2_S, when the butane concentration exceeds 800 ppm in the air, it causes dizziness, syncope, and nausea in humans. Fan et al. stated that the mixture of gas (1.6–8.5%) of butane and air can cause an explosion [5]. So, the detection of butane is essential for human safety.

Semiconductor metal oxides (SMOs) have been strongly suggested and extensively studied as gas sensors because of their advantages, such as simple fabrication, real-time sensing, low cost, portability, and ability to synthesize different nanocomposites [6]. Conventional SMOs have abundant oxygen vacancies, which provide extra active sites for enhancing gas-sensing properties [7]. Cobalt-containing spinel oxides (ACo_2_O_4_, A = Ni, Zn, Cu) are an emerging class of ternary SMOs, owing to their widespread attention in different research areas such as gas sensors [8,9], super-capacitors [10], lithium-ion batteries [11], photocatalysis [12], etc. Among different spinel oxides, NiCo_2_O_4_, a typical p-type material, has found interesting applications in the field of gas sensors [13]. NiCo_2_O_4_ has the oxidation states of Ni (Ni^3+^/Ni^2+^) and Co (Co^3+^/Co^2+^) in most studies, and Ni immerses 16d octahedral sites, while Co is assorted in 16d octahedral as well as 8a tetrahedral sites [14]. Du et al. stated the gas-sensing properties of NiCo_2_O_4_ hollow microtubules and found that a sensor of NiCo_2_O_4_ detected 100 ppm xylene at the high operating temperature of 220 °C, and the response was 9.2 [15]. NiCo_2_O_4_ was used to study the gas-sensing properties of n-butanol, xylene, and H_2_S [16,17,18]. Dang et al. also detected 100 ppm n-butanol gas at a temperature of 165 °C in layered nanoflower-like NiCo_2_O_4_ [16]. However, the above-discussed metal oxide-based gas sensor was found to have poor selectivity, high operating temperature, etc. [19,20]. Thus, a gas sensor based on a nanocomposite of 2D material and pure metal oxide is necessary to detect hazardous gases at lower temperatures.

A member of 2D layered materials, MXenes (M_n+1_X_n_T_x_, where M represents the transition metals including Ti, Nb, Zr, Cr, etc.; X stands for C or N; and T_x_ is designated for terminated functional groups such as fluorine (–F), hydroxyl (–OH), or oxygen (–O)) have been used for outstanding achievements in different fields, such as gas sensors [21,22,23,24,25], energy storage devices [26], super-capacitors [27], and humidity sensing [28]. Among the MXene family, Ti_3_C_2_T_x_, which was discovered in 2011, comprises transition metal carbides and nitrides that have received great attraction from researchers because of their relatively low density (4.91 g/cm^−3^) [29], sizeable BET-specific surface area, unique tunable electronic structure, excellent hydrophilicity features, and abundant surface terminals [30]. Critically, MXene maintains excellent metallic conductivity during surface modifications [31], which makes MXene different from other 2D materials such as graphene, MoS_2_, WS_2_, etc. More importantly, the distinct character of MXenes is that, with their surface modifications, their excellent metallic conductivity is not sacrificed. For example, Kim et al. stated that the signal-to-noise ratio of Ti_3_C_2_T_x_ is two times higher than that of traditional 2D materials (MoS_2_, graphene, black phosphorus, etc.) [31,32]. Various examples from the literature based on Ti_3_C_2_T_x_ and metal oxides were studied to highlight the impact of Ti_3_C_2_T_x_ as gas sensors. Pasupuleti et al. and Wang et al. synthesized different nanocomposites with MXene, such as CuO/Ti_3_C_2_T_x_ MXene and SnO-SnO_2_/Ti_3_C_2_T_x_; these sensors improved the gas sensing properties five times more than those of pure CuO sensors and SnO-SnO_2_ sensors [25,33]. Due to the enhanced gas sensing properties of MXene-based nanocomposites, it can be expected that a nanocomposite of MXene Ti_3_C_2_T_x_ and NiCo_2_O_4_ could improve gas sensing properties. Until now, there have been rare instances in the literature reporting H_2_S sensing using MXene Ti_3_C_2_T_x_ and NiCo_2_O_4_ nanocomposites.

Herein, four different nanocomposites based on MXene Ti_3_C_2_T_x_/NiCo_2_O_4_, Ti_3_AlC_2_/NiCo_2_O_4_, WS_2_/NiCo_2_O_4_, and MoSe_2_/NiCo_2_O_4_ were synthesized using the hydrothermal method. Supportive characterizations such as XRD, SEM, TEM, HRTEM, BET, FTIR, and XPS were performed to check the microstructure and morphology of the nanocomposites. The sensor chips were synthesized with nanocomposites and gas-sensing properties were analyzed. The response (S) was defined as the ratio of the resistance in target gas Rg and resistance in air Ra, S = R_g_/R_a_. The sensor of MXene/NiCo_2_O_4_ detected the highest responses toward H_2_S and butane at room temperature and 90 °C, respectively. Significantly fewer responses to all gases were detected using other sensor sheets, suggesting the better selectivity of MXene/NiCo_2_O_4_ toward H_2_S and butane; additionally, the sensor of MXene/NiCo_2_O_4_ also showed minimum responses to 0.1 ppm H_2_S and 5 ppm butane, respectively. The current work provides a broad perspective on the application of H_2_S sensors.

## 2. Experimental Results and Discussion

### 2.1. Morphology and Structure of Products

**Figure 1:**
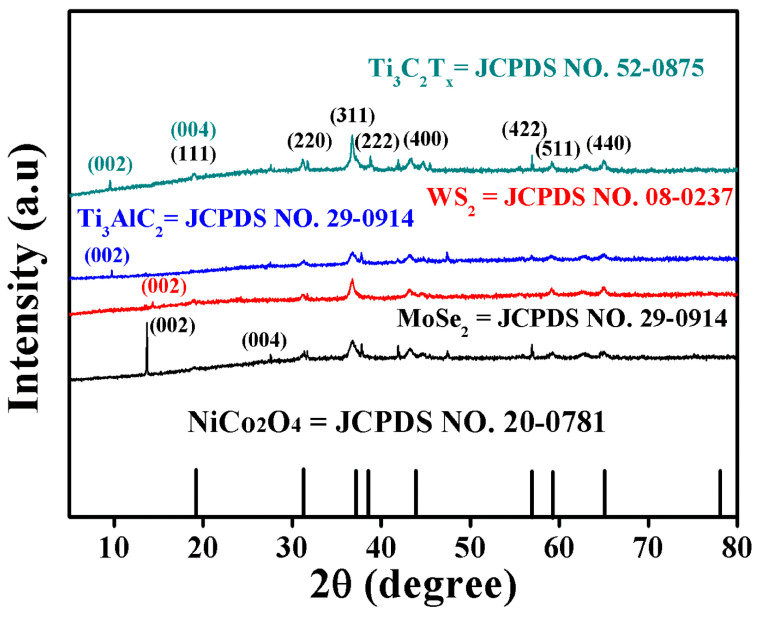
XRD patterns of MXene Ti_3_C_2_T_x_/NiCo_2_O_4_, Ti_3_AlC_2_/NiCo_2_O_4_, WS_2_/NiCo_2_O_4_, and MoSe_2_/NiCo_2_O_4_.

In Figure 1, XRD diffraction peaks were performed to find the structural information of MXene Ti_3_C_2_T_x_/NiCo_2_O_4_, Ti_3_AlC_2_/NiCo_2_O_4_, WS_2_/NiCo_2_O_4_, and MoSe_2_/NiCo_2_O_4_. The XRD patterns of all products stated that the diffraction peaks located at the 2θ values of 18.90°, 31.14°, 36.59°, 38.40°, 44.62°, 55.43°, 59.09°, and 64.98° consisted of (111), (220), (311), (222), (400), (422), (511), and (440) planes of NiCo_2_O_4,_ respectively. Small peaks of Ti_3_C_2_T_x_, Ti_3_AlC_2_, WS_2_, and MoSe_2_ were also found, and no other peaks were revealed, suggesting the successful synthesis of designed nanocomposites. The estimated crystallite sizes of NiCo_2_O_4_ were 13.36, 10.86, 11.4, and 11.5 nm based on the (311) peak in the nanocomposites of MXene Ti_3_C_2_T_x_/NiCo_2_O_4_, Ti_3_AlC_2_/NiCo_2_O_4_, WS_2_/NiCo_2_O_4_, and MoSe_2_/NiCo_2_O_4_, respectively.

**Figure 2:**
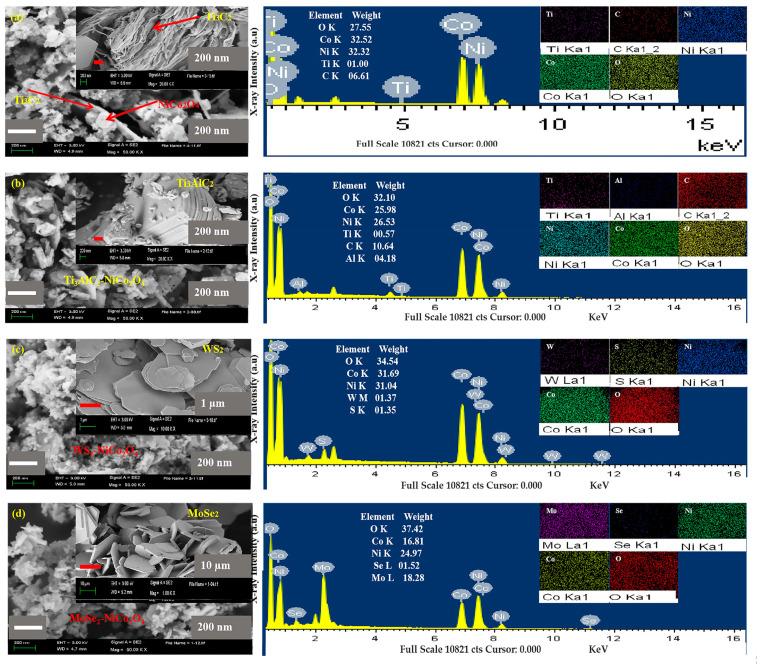
SEM images and EDS (spectrum/scattering) of MXene/NiCo_2_O_4_ (**a**), Ti_3_AlC_2_/NiCo_2_O_4_ (**b**), WS_2_/NiCo_2_O_4_ (**c**), and MoSe_2_/NiCo_2_O_4_ (**d**).

To find the morphologies of the synthesized samples, SEM images were introduced. Figure 2a–d shows the SEM images and EDS spectra (mappings) of MXene/NiCo_2_O_4_, Ti_3_AlC_2_/NiCo_2_O_4_, WS_2_/NiCo_2_O_4_, and MoSe_2_/NiCo_2_O_4_. Spherical NiCo_2_O_4_ nanoparticles with an average particle size around 30–40 nm were found in all the products and clear layered structure of MXene. SEM confirmed the attachment of spherical NiCo_2_O_4_ nanoparticles with the layered structure of MXene. The EDS spectra and scattering of each element also verified the successful synthesis of the synthesized nanocomposites.

**Figure 3:**
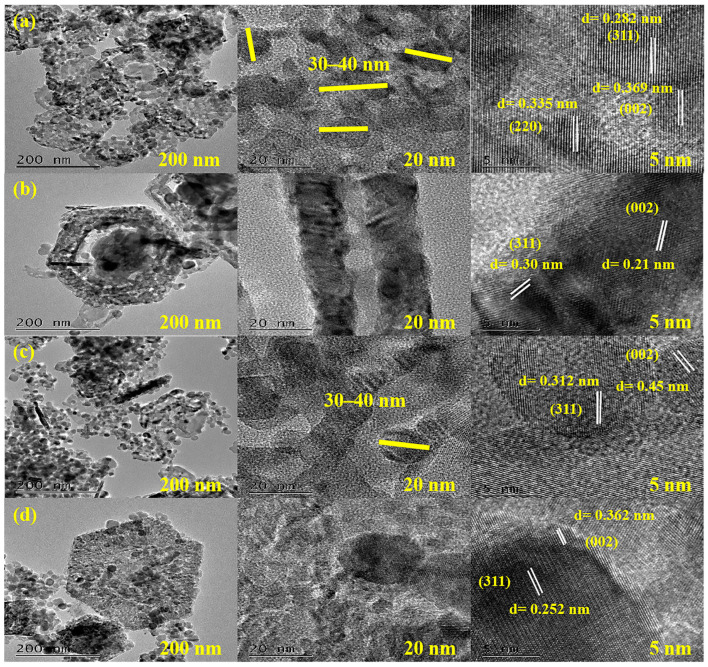
TEM and HRTEM images of MXene/NiCo_2_O_4_ (**a**), Ti_3_AlC_2_/NiCo_2_O_4_ (**b**), WS_2_/NiCo_2_O_4_, (**c**) and MoSe_2_/NiCo_2_O_4_ (**d**).

The TEM and HRTEM images also described the morphology and particle size of the samples. Figure 3a–d shows different TEM and HRTEM images for MXene/NiCo_2_O_4_, Ti_3_AlC_2_/NiCo_2_O_4_, WS_2_/NiCo_2_O_4_, and MoSe_2_/NiCo_2_O_4_. The clear particle size of NiCo_2_O_4_ of about 30–40 nm was checked in all nanocomposites. Figure 3a shows the lattice stripe spacing of 0.829 nm, 0.335 nm, and 0.369 nm, corresponding to the (311), (220), and (002) planes of NiCo_2_O_4_ and MXene. Figure 3b shows clear spacing of 0.30 nm and 0.21 nm, related to the (311) and (002) planes of NiCo_2_O_4_ and Ti_3_AlC_2_. Figure 3c,d shows the clear spacing of 0.312 nm and 0.45 nm for the (311) and (002) planes of NiCo_2_O_4_ and WS_2_, while the spacing of 0.252 nm and 0.362 nm correspond to the (311) and (002) planes of NiCo_2_O_4_ and MoSe_2._

**Figure 4:**
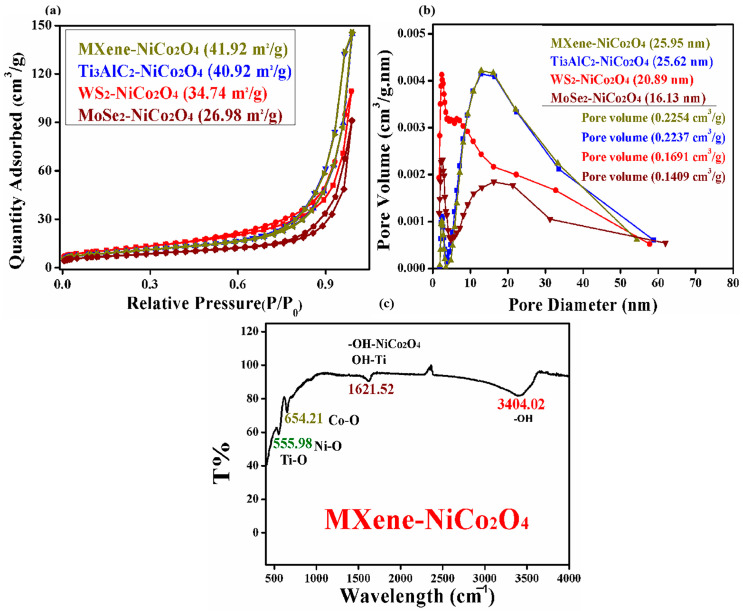
N_2_ adsorption–desorption isotherms and pore size distributions of MXene/NiCo_2_O_4_, Ti_3_AlC_2_/NiCo_2_O_4_, WS_2_/NiCo_2_O_4_, and MoSe_2_/NiCo_2_O_4_ (**a**,**b**) and the FTIR spectrum of MXene/NiCo_2_O_4_ (**c**).

N_2_ adsorption–desorption experiments were performed, as shown in Figure 4a,b, to study the BET-specific area and pore size distribution of the synthesized products including MXene/NiCo_2_O_4_, Ti_3_AlC_2_/NiCo_2_O_4_, WS_2_/NiCo_2_O_4_, and MoSe_2_/NiCo_2_O_4_. The data showed that the BET-specific surface areas and pore sizes of MXene/NiCo_2_O_4_, Ti_3_AlC_2_/NiCo_2_O_4_, WS_2_/NiCo_2_O_4_, and MoSe_2_/NiCo_2_O_4_ were 41.92, 40.92, 34.74, and 26.98 m^2^/g, and 25.95 nm, 25.62 nm, 20.89 nm, and 16.13 nm, respectively. The pore volumes of MXene/NiCo_2_O_4_, Ti_3_AlC_2_/NiCo_2_O_4_, WS_2_/NiCo_2_O_4_, and MoSe_2_/NiCo_2_O_4_ were 0.2254 cm^3^/g, 0.2237 cm^3^/g, 0.1691 cm^3^/g, and 0.1409 cm^3^/g, respectively. The type IV hysteresis loop was determined from the N_2_ adsorption–desorption isotherm, indicating the porous characteristic of spherical NiCo_2_O_4_. The BET surface area and pore size of MXene/NiCo_2_O_4_ are greater than other nanocomposites, suggesting that porous structures offer extra tunnels that stimulate the penetration of gas molecules into the sensitive particle and surface reaction, which in turn increase test gas adsorption as well as enhance the gas sensing properties of MXene/NiCo_2_O_4_ [34]. The FTIR spectrum of MXene/NiCo_2_O_4_ is shown in Figure 4c. The vibration peak cited at 3404.02 cm^−1^ corresponded to the hydrogen-bonded OH or coordinated H_2_O. The peaks at the values of 555.98 cm^−1^ and 1623 cm^−1^ were related to the bending vibrations (OH–Ti) and (Ti–O), suggesting oxygen-containing functional groups on the surface of MXene [35,36]. The adsorption peaks at the values of 555.98 cm^−1^ and 654.21 cm^−1^ corresponded to the bonding formation of Ni–O and Co–O in NiCo_2_O_4_ material [37].

**Figure 5:**
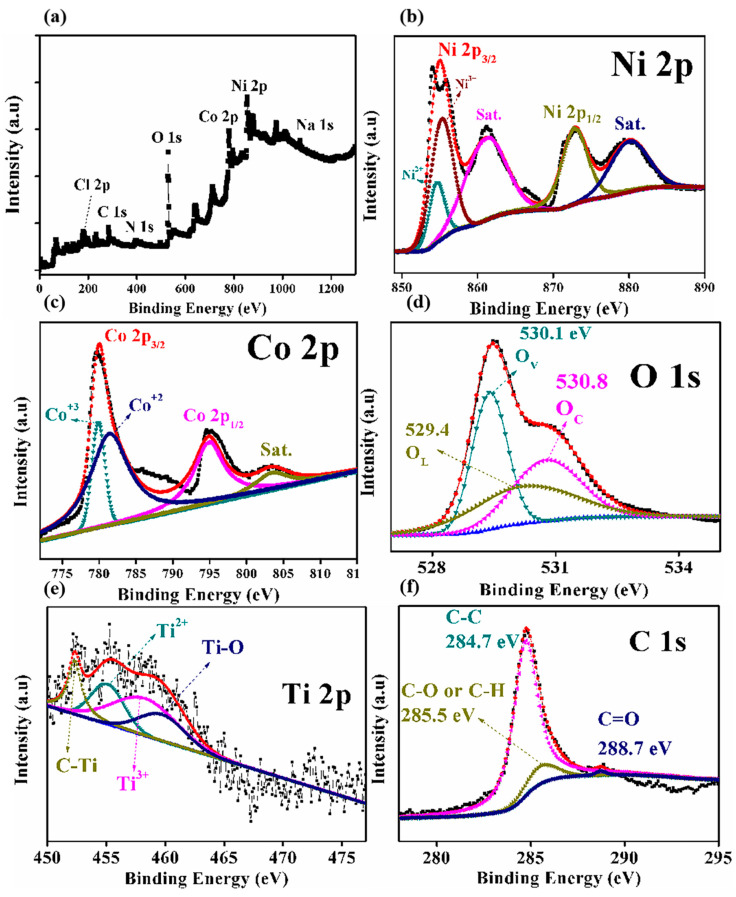
XPS spectra of the full spectrum (**a**), Ni 2p (**b**), Co 2p (**c**), O 1s (**d**), (Ti 2p (**e**), and C 1s (**f**) spectra of MXene/NiCo_2_O_4_.

In Figure 5, the surface chemical composition and electronic state of the nanocomposite MXene/NiCo_2_O_4_ were studied using X-ray photoelectron spectra characteristics. The full XPS spectrum is shown in Figure 5a, which suggests the presence of all the elements. The spectrum of Ni 2p is shown in Figure 5b, which specifies two peaks corresponding to Ni 2p_3/2_ and Ni 2p_1/2_, respectively, while the main spin-orbit doublets Ni 2p_3/2_ were further divided into Ni^3+^ and Ni^2+^ [38]. Two satellite peaks were found as well. The spectrum of Co 2p is shown in Figure 5c, which shows two peaks at values of 779.9 eV and 794.9 eV, which are related to Co 2p_3/2_ and Co 2p_3/2_, respectively. The main peak of Co 2p_3/2_ was further split into two Co^3+^ and Co^2+^, and a satellite peak was also detected [39]. Figure 5d shows the O 1s spectra of Ti_3_C_2_/NiCo_2_O_4_, which states that the O 1s spectrum was divided into three oxygen species (O_L_, O_V_, and O_C_). The Ti 2p spectrum of MXene/NiCo_2_O_4_ showed four peaks at 452.3, 455.1, 458.2, and 459.7 eV, which are related to C–Ti, Ti^2+^, Ti^3+^, and Ti–O, respectively [40]. The C 1s spectrum showed three peaks (C–C, C–O or C–H, C=O) at the values of (284.7 eV, 285.5 eV, and 288.7 eV), respectively [41].

### 2.2. Gas-Sensing Properties

**Figure 6:**
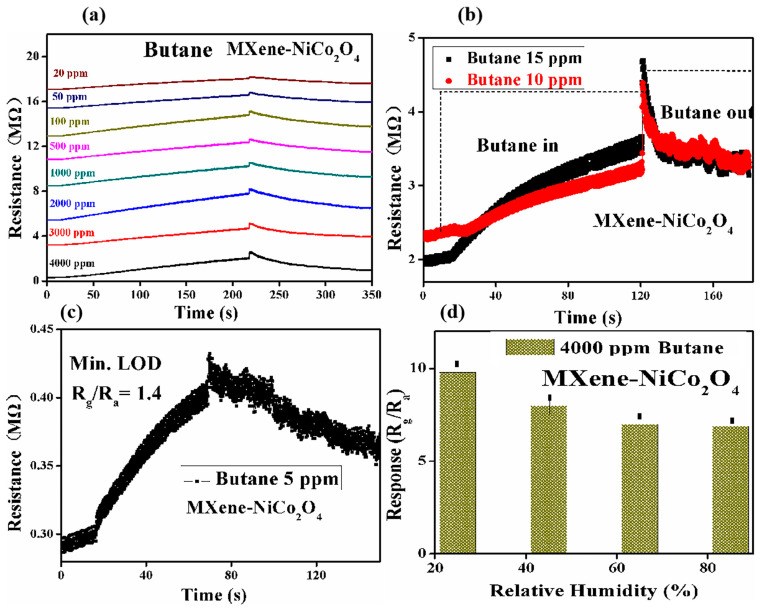
Resistance changes in the MXene/NiCo_2_O_4_ composite-based gas sensor for different concentrations of butane at 90 °C (**a**–**c**) and the relationship between the response of MXene/NiCo_2_O_4_ and different RHs at 90 °C (**d**).

The gas-sensing properties of four different sensor sheets were studied, and the sensors were fabricated with the synthesized materials such as MXene/NiCo_2_O_4_, Ti_3_AlC_2_/NiCo_2_O_4_, WS_2_/NiCo_2_O_4,_ and MoSe_2_/NiCo_2_O_4_. The gas sensor of MXene/NiCo_2_O_4_ detected the highest responses toward H_2_S and butane at different operating temperatures compared with the other sensors. In Figure 6a–c, the gas sensor based on MXene/NiCo_2_O_4_ shows that the responses to 4000 ppm, 3000 ppm, 2000 ppm, 1000 ppm, 500 ppm, 100 ppm, 50 ppm, 20 ppm, 15 ppm, 10 ppm, and 5 ppm butane were 9.8, 8.8, 7.4, 6.6, 5.2, 4.6, 3.8, 2.9, 2.1, 1.9, and 1.2, respectively, at the operating temperature of 90 °C. The minimum detection limit was 5 ppm. The response/recovery times for 4000 ppm butane were ~200/~180 s, and the response/recovery times for 3000 ppm, 2000 ppm, 1000 ppm, 500 ppm, 100 ppm, 50 ppm, 20 ppm, 15 ppm, 10 ppm, and 5 ppm butane were 199 s/35 s, 190 s/36 s, 195 s/40 s, 200 s/42 s, 201 s/34 s, 192 s/32 s, 189 s/32 s, 198 s/38 s, 195 s/38 s, and 197 s/32 s, respectively. The results showed that the response was improved by increasing the concentrations of butane. Figure 6d shows that the responses of the MXene/NiCo_2_O_4_-based gas sensor were decreased with the enhancement in relative humidity (RH); the attachment of water molecules on the surface of the sensing material at higher RH might be the reason for the decreasing response.

**Figure 7:**
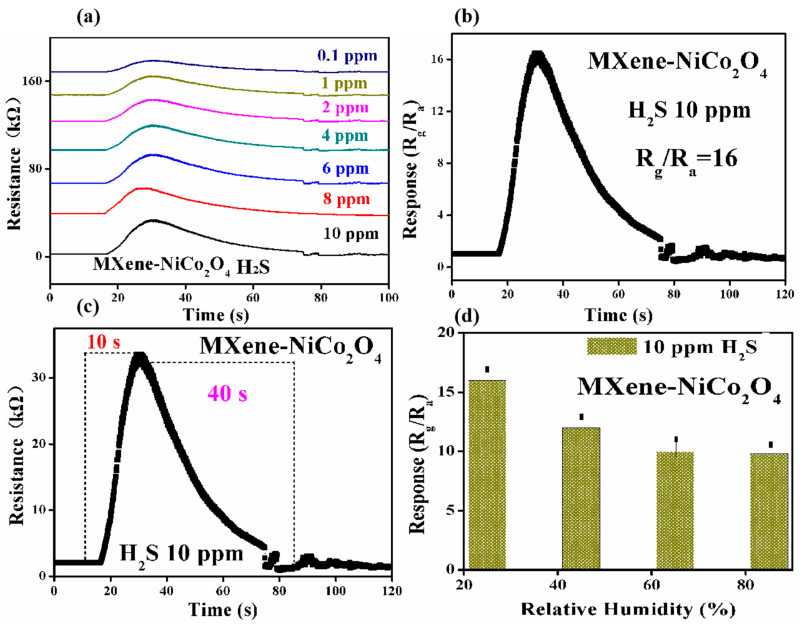
Resistance changes in the MXene/NiCo_2_O_4_ composite-based gas sensor at different concentrations of H_2_S at room temperature (**a**–**c**) and the relationship between the response of MXene/NiCo_2_O_4_ and different RH at room temperature (**d**).

Figure 7a–c shows that the gas sensor of MXene/NiCo_2_O_4_ yielded responses such as 16, 13, 10, 8, 4, 2.4, and 1.8 to 10 ppm, 8 ppm, 6 ppm, 4 ppm, 2 ppm, 1 ppm, and 0.1 ppm, H_2_S, respectively, at room temperature. The minimum detection limit was 0.1 ppm. The response/recovery times for 10 ppm H_2_S were 10 s/40 s, respectively, while the response/recovery times for 8 ppm H_2_S, 6 ppm H_2_S, 4 ppm H_2_S, 2 ppm H_2_S, 1 ppm H_2_S, and 0.1 ppm H_2_S were 9 s/38 s, 10 s/39 s, 8 s/41 s, 9 s/42 s, 8 s/40 s, and 7 s/36 s, respectively. With the enhancement in H_2_S concentration, the response was increased. Figure 7d describes the relationship between the response of the MXene/NiCo_2_O_4_ composite-based gas sensor and relative humidity (RH). The results demonstrated that when the RH was increased in the test chamber, by values such as 45%, 65%, and 85%, the response decreased.

**Figure 8:**
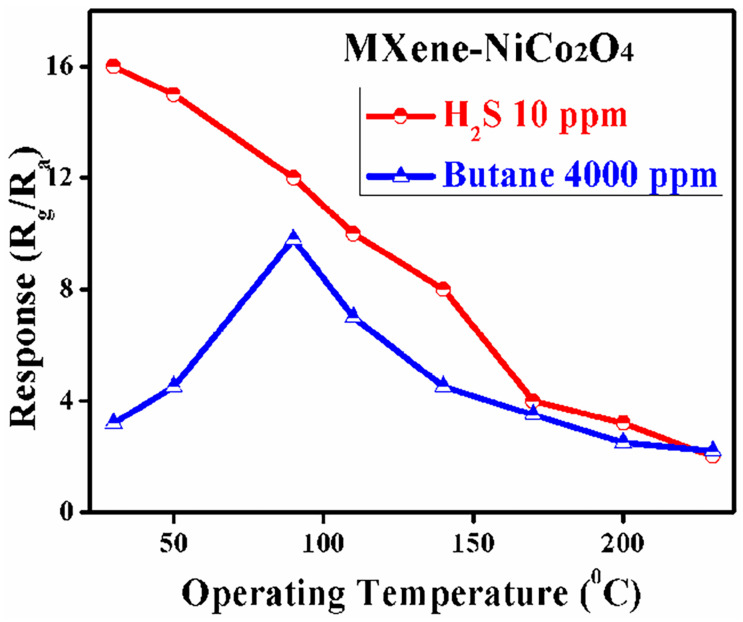
Responses of MXene/NiCo_2_O_4_ to 10 ppm H_2_S and 4000 ppm butane at different operating temperatures.

Figure 8 shows the responses of the gas sensors based on spherical MXene/NiCo_2_O_4_ for 10 ppm H_2_S and 4000 ppm butane at different operating temperatures, which shows the relationship between the responses of the sensor based on MXene/NiCo_2_O_4_ and the operating temperatures. Sun et al. stated that the operating temperature affects the species of adsorption oxygen, the carrier concentration/resistance, and response–recovery time [42]. The sensor sheet depicted a high response toward 10 ppm H_2_S at room temperature and 4000 ppm butane at 90 °C, and the increased operating temperature decreased the response. The results showed that the operating temperature of 90 °C was the optimal temperature for butane sensing and that room temperature was optimal for H_2_S sensing. The highest response may relate to the highest BET surface area, the p–p heterojunction between Ti_3_C_2_T_x_ and NiCo_2_O_4_, and abundant active sites. H_2_S-sensing and butane-sensing properties have been studied by many researchers, who suggested that in the gas sensing of H_2_S/butane based on the reaction of H_2_S/butane and adsorbed oxygen on the surface of NiCo_2_O_4_, at low temperatures, oxygen adsorbs on the surface of sensitive material and captures extra electrons from the conduction band.

**Figure 9:**
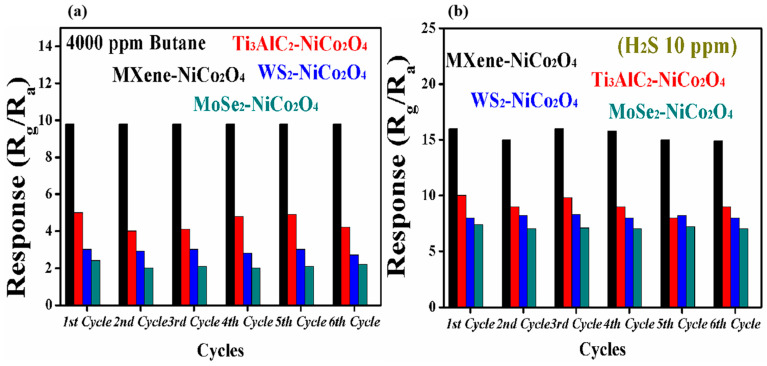
The reproducibility of the gas sensors based on MXene/NiCo_2_O_4_, Ti_3_AlC_2_/NiCo_2_O_4_, WS_2_/NiCo_2_O_4_, and MoSe_2_/NiCo_2_O_4_ to 4000 ppm butane at 90 °C (**a**) and 10 ppm H_2_S at room temperature (**b**).

Figure 9a,b shows the reproducibility of the MXene/NiCo_2_O_4_, Ti_3_AlC_2_/NiCo_2_O_4_, WS_2_/NiCo_2_O_4_, and MoSe_2_/NiCo_2_O_4_ gas sensors to 10 ppm H_2_S and 4000 ppm butane at room temperature and at the operating temperature of 90 °C. The responses of the MXene/NiCo_2_O_4_ primary sensor to 10 ppm H_2_S dropped slightly, but for butane, it was quite stable. Overall, the sensor showed good reproducibility for both gases compared with the other sensors.

**Figure 10:**
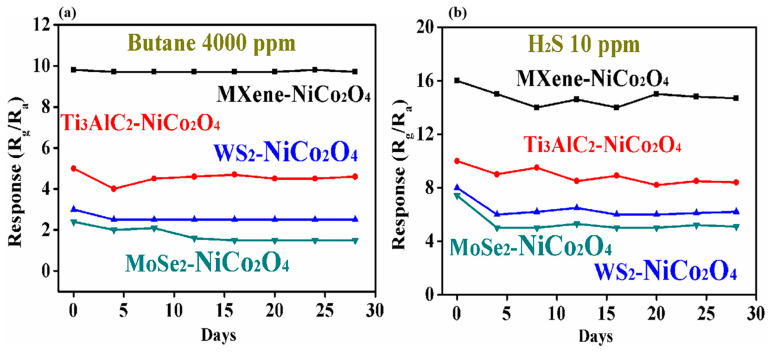
The stability of the gas sensors based on MXene/NiCo_2_O_4_, Ti_3_AlC_2_/NiCo_2_O_4_, WS_2_/NiCo_2_O_4_, and MoSe_2_/NiCo_2_O_4_ to 4000 ppm butane at 90 °C (**a**) and 10 ppm H_2_S at room temperature (**b**).

Long-term stability tests are also an essential factor in gas sensors. The long-term stabilities of the gas sensors based on MXene/NiCo_2_O_4_, Ti_3_AlC_2_/NiCo_2_O_4_, WS_2_/NiCo_2_O_4_, and MoSe_2_/NiCo_2_O_4_ to 4000 ppm butane at 90 °C and 10 ppm H_2_S at room temperature are shown in Figure 10a,b. The responses of the MXene/NiCo_2_O_4_-based gas sensor for 4000 ppm butane were quite stable, but the response for 10 ppm H_2_S dropped slightly by 16 to 14 in the first 10 days, but after that, it was stable, implying the better stability of the sensor.

**Figure 11:**
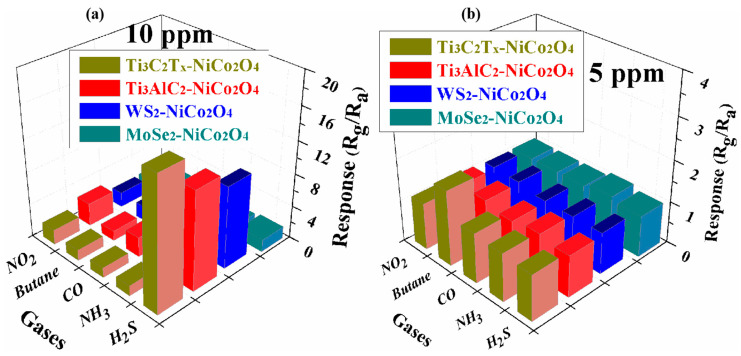
The cross-selectivity of the MXene/NiCo_2_O_4_-, Ti_3_AlC_2_/NiCo_2_O_4_-, WS_2_/NiCo_2_O_4_- and MoSe_2_/NiCo_2_O_4_-based gas sensors for 10 ppm gases at room temperature (**a**) and for 5 ppm gases at 90 °C (**b**).

The gas sensors based on MXene/NiCo_2_O_4_, (b) Ti_3_AlC_2_/NiCo_2_O_4_, (c) WS_2_/NiCo_2_O_4_, and (d) MoSe_2_/NiCo_2_O_4_ showed responses toward the different gases at room temperature, as shown in Figure 11a, and at 90 °C, as shown in Figure 11b. These graphs demonstrate that the sensor of MXene/NiCo_2_O_4_ showed good selectivity while testing different gases of 10 ppm at room temperature and testing different gases of 5 ppm at the operating temperature of 90 °C, respectively. Various gas sensors for H_2_S and butane sensing were studied. As listed in Table 1, the sensor fabricated with MXene/NiCo_2_O_4_ showed a high response, short response/recovery time, and minimum limit of detection at room temperature and the operating temperature of 90 °C.

**Figure 12:**
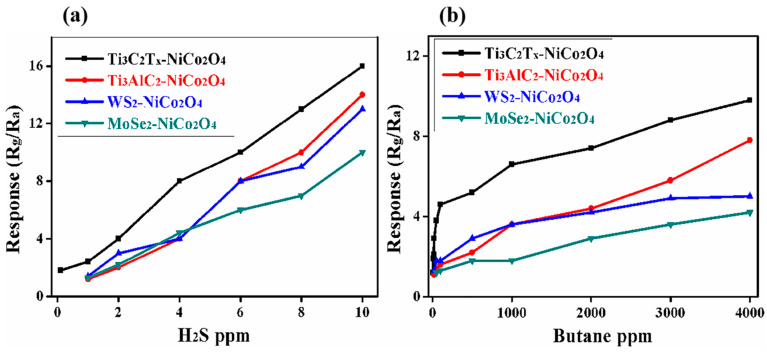
Relationship between the response of MXene/NiCo_2_O_4_ and H_2_S concentrations at room temperature (**a**) and the relationship between the response of MXene/NiCo_2_O_4_ and butane concentrations at 90 °C (**b**).

Figure 12a,b shows that the responses increased with the increase in H_2_S concentration and butane concentration, indicating that the sensors showed a linear relationship between their responses and the H_2_S/butane concentrations. The gas sensor of MXene/NiCo_2_O_4_ showed linearity between its responses and the concentrations of H_2_S/butane, which showed the promising applicability of the current sensor.

### 2.3. Gas-Sensing Mechanism

**Figure 13:**
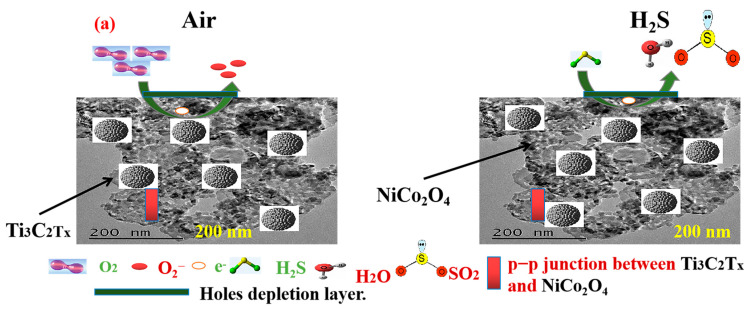

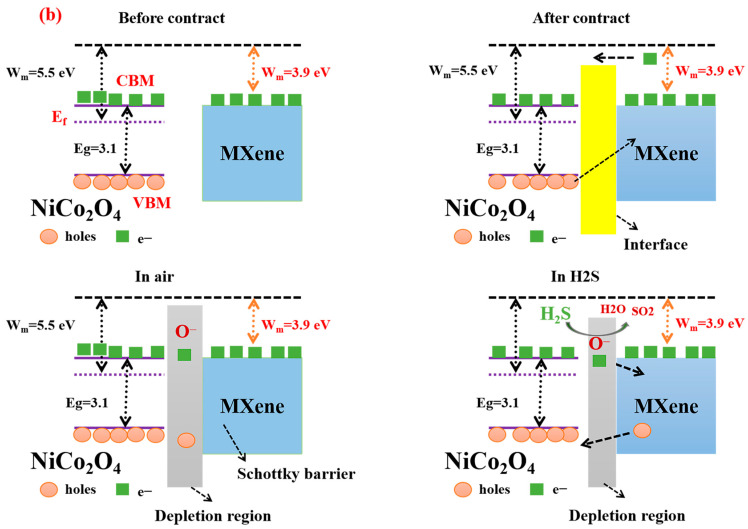
Gas sensing mechanism and energy band diagram of MXene/NiCo_2_O_4_ (**a**,**b**).

The typical p-type NiCo_2_O_4_ is widely used as gas sensors, although Ti_3_C_2_T_x_ MXene also displayed p-type sensing behavior in our case [43]. So, the p–p hetero-junction based on the nanocomposite of MXene/NiCo_2_O_4_ showed the p-type gas sensing mechanism. The mechanism is based on the chemical reaction between the target gas and chemisorbed oxygen on the sensitive material [44]. The mechanism was similar for H_2_S and butane in the current manuscript, so it is explained for H_2_S only. The hypothesized gas-sensing mechanism of MXene/NiCo_2_O_4_ is shown in Figure 13. When the MXene/NiCo_2_O_4_-based gas sensor was introduced into the air, the oxygen molecules captured electrons from sensing material and generated O_2_^−^ (less than 100 °C) (Equations (1)–(4)), this whole process created hole accumulation layers (HALs), which reduced the resistance of the sensing material. After HAL creation, the sensor was placed into H_2_S; the electrons were released back into the conduction band of the sensing mechanism with the existence of SO_2_ and 2H_2_O, thus making the HAL thinner, increasing the height of the Schottky barrier, and resulting in an enhancement in resistance.

O_2 (gas)_ → O_2 (ads.),_ (1)

O_2 (ads.)_ + e^−^ → O_2_^−^_(ads.),_ T < 100 °C (2)

O_2_^−^_(ads.)_ + e^−^ → 2O^−^_(ads.),_ (3)

O^−^_(ads.)_ + e^−^ → O^2−^_(ads.),_ (4)

After HAL creation, the sensor was placed into H_2_S. The electrons were then released back into the conduction band of sensing mechanism with the existence of SO_2_ and 2H_2_O (Equation (5)), thus making the HAL thinner, increasing the height of the Schottky barrier, and resulting in an enhancement in resistance. The reaction was as follows.

2H_2_S (g) + 2O_2_^−^ (ads) → 2SO_2_ + 2H_2_O + 3e^−^ (5)

The mechanism was basically based on the adsorption/desorption mechanism and oxidation/reduction mechanism. The charge carrier transfer also played a critical role in the gas-sensing properties. The gas-sensing mechanism was followed by one of the previous studies [23]. As it is well-known, MXene plays a p-type sensing behavior and NiCo_2_O_4_ also shows p-type behavior, so the sensing behavior would be typical p-type in this study, and the response was calculated as S = R_g_/R_a_. A Schottky barrier formed at the p–p heterojunction interface to equalize the Fermi level due to the transfer of carriers. The work function of NiCo_2_O_4_ (5.5 eV) was higher than Ti_3_C_2_T_x_ MXene (3.9 eV) [45,46]. The electrons flow from MXene to NiCo_2_O_4_ and holes from NiCo_2_O_4_ to MXene; this reaction would reduce the depletion layer as well as enhance the resistance of the sensitive material. The band gap of p-type NiCo_2_O_4_ was approximately 3.1 eV, which was higher than MXene (1.1 eV) [47]. Another factor that enhances the gas sensing properties of MXene/NiCo_2_O_4_ is the abundant functional groups present in MXene, as proved with XPS such as (-O, -Cl, and -Na), which also enhanced active sites as well as oxygen adsorption sites [48]. High carrier mobility and electrical conductivity of MXene also improved the gas sensing properties of MXene/NiCo_2_O_4_. The highest BET surface area of MXene/NiCo_2_O_4_ corresponded to the adsorption of oxygen species and H_2_S, resulting in enhanced absorption and diffusion capacity of H_2_S molecules.

**Table 1:** Comparison of different gas sensors.

Synthesis Materials	Operating Temp. (°C)	H_2_S/Propane ppm	Response	LOD	Res./Rec. Times	Ref.
NFO nanoparticles	150 (°C)	200 ppm/H_2_S	1.75	NA	NA	[49]
NiO@ZnO nanotubes	215 (°C)	50 ppm/H_2_S	474	1 ppm	50/124 s	[50]
Fe_2_O_3_/NiO nanoplate	300 (°C)	200 ppm/H_2_S	1.9	5 ppm	NA	[51]
NiO thin films	300 (°C)	8 ppm/H_2_S	1.46	NA	48/24 min	[52]
NiFe_2_O_4_-MWCNT	300 (°C)	100 ppm/H_2_S	2.5	NA	110/NA	[53]
Fe_2_O_3_/NiO	200 (°C)	10 ppm/H_2_S	8	NA	100/20 s	[54]
α-Fe_2_O_3_/MoSe_2_	room temp.	30 ppm/H_2_S	57.7	1 ppm	50/53 s	[55]
MgFe_2_O_4_ pellets	425 (°C)	2000 ppm/butane	3.45	NA	63/178 s	[56]
Pt-Zn_2_SnO_4_-ZnO nanorods	250 (°C)	9000 ppm/LPG	NA	1000 ppm	NA	[57]
ZnO thin film	380 (°C)	1660 ppm/butane	2.33	NA	~340/~230 s	[58]
MXene/NiCo_2_O_4_	room temp. 90 (°C)	10 ppm/H_2_S 4000 ppm/butane	16 9.8	0.1 ppm 5 ppm	10/40 s ~200/~180 s	This work

## 3. Experimental Section

### 3.1. Materials

All the chemicals used in the synthesis method were bought from Sinopharm Chemical Reagent Co., Ltd. (Shanghai, China). The materials such as titanium aluminum carbide (Ti_3_AlC_2_, MAX), hydrochloric acid (HCL, 38%), ammonium fluoride (NH_4_F), tungsten disulfide (WS_2_), molybdenum diselenide (MoSe_2_), nickel chloride pentahydrate (SnCl_2_·5H_2_O), tin chloride pentahydrate (SnCl_2_·5H_2_O), and sodium hydroxide (NaOH) were utilized in the synthesis method without further purification.

### 3.2. Synthesis of Layered Ti_3_C_2_T_x_

In total, 20 mL of hydrochloric acid (HCL, 38%) was poured into a 100 mL PTFE beaker. Then, 2.96 g NH_4_F was added to it while magnetically stirring for 30 min until the solution was uniformly transparent. After that, 0.5 g of Ti_3_AlC_2_ was slowly added above the suspension, and the stirring was continued. After three days, the mixture was washed many times with ethanol and water in centrifuge tubes at 8500 rpm until the supernatant reached a pH of approximately 7, and then it was dried for 5 h at 80 °C in an oven.

### 3.3. Synthesis of Materials

**Figure 14:**
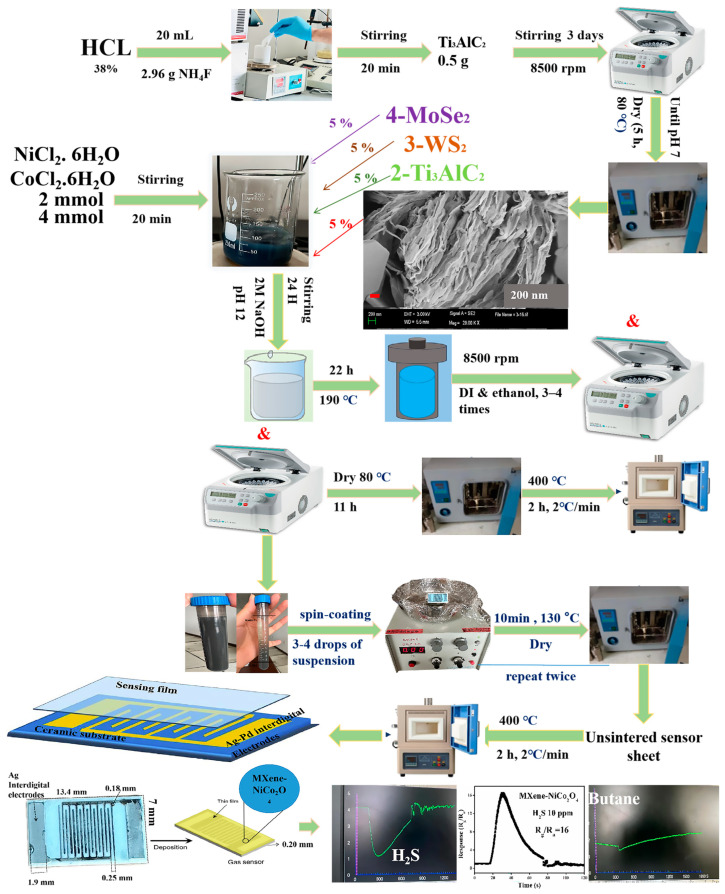
The synthesis method and the sensor fabrication method.

The synthesis of nanocomposites is described in Figure 14. The details were as follows: NiCl_2_. 6H_2_O (2 mmol) and CoCl_2_. 6 H_2_O (4 mmol) were added into four beakers. In the black suspension, 5% of different 2D materials such as Ti_3_C_2_T_x_, Ti_3_AlC_2_, WS_2_, and MoSe_2_ were added into the suspensions during stirring; after 5–10 min, 2M NaOH was added into the suspensions to adjust the pH to 12. Subsequently, after 24 h of stirring, the solutions were transferred into 50 mL stainless steel autoclaves, and the oven was adjusted to 22 h and 190 °C. After the autoclave process, the samples were washed 3–4 times with DI water and ethanol using centrifugation (8500 rpm). After this stage, the samples were divided into two parts: one with a solid and liquid mixture was used to fabricate sensor sheets, and the second was dried in an oven for 11 h and 80 °C. The final step was the calcination process at 400 °C for 2 h and 2 °C/min.

### 3.4. Fabrication of Sensor Sheets

As discussed above, one part of the samples (the mixture of solid and liquid) after the autoclave process was used to fabricate sensor sheets. The sensor sheet was bought from Wei Sheng Electronics, Zhenjiang, Jiangsu, China. The alumina substrate of the sensor sheet had a thickness of 0.20 mm with two Ag inter-digital electrodes (1.9 mm width). The length and width of the sensor sheet were 13.4 mm and 7 mm, respectively. Moreover, the sensor sheet had a total of 16 inter-digits; the thickness of a single digit was 0.18 mm, and the distance between the two digits was 0.25 mm. The details were as follows: the ceramic sensor chip was fixed on a spin coating machine, the suspension liquid in the test tube was shaken evenly, a small amount of the suspension liquid was drawn with a disposable dropper, and 4~5 drops were dropped on the side of the ceramic negative with a cross-fingered electrode to spin the coating into a film. After that, the coated sensor sheet was placed into a glass dish and put in an oven at 130 °C for 10 min. The purpose was to volatilize the alcohol and dry the sensor sheet. After drying twice, the dried sensor sheet was put into a crucible and placed in a muffle furnace for sintering. The temperature was 400 °C, the duration was 2 h, and the heating rate was 2 °C/min. It naturally cooled to room temperature to obtain the hetero-structure thin film sensor sheet for detecting different gases in this paper.

### 3.5. Physical Characterization of Materials

The synthesized samples were characterized with different characterizations such as X-ray diffraction (XRD, D/MAX-Ultima with a Cu Kα source, 2°/min scanning rate and scanning angle from 10° to 80°, and a power of 40 kV and 40 mA; Rigaku, Tokyo, Japan), scanning electron microscopy (SEM, ZEISS Gemini 500, Carl Zeiss AG, Oberkochen, Germany) with the component of energy dispersive spectroscopy (EDS), transmission electron microscopy (TEM, JEM-3200FS, JEOL, Tokyo, Japan), high-resolution transmission electron microscopy (HRTEM, JEM-2100F, JEOL, Tokyo, Japan), Brunauer–Emmett–Teller (BET ASAP2010C instrument, Norcross, GA, USA), Fourier transform infrared (a Nicolet 6700 FTIR spectrometer in the range of 400–4000 cm^−1^ with the KBr pellet technique, FTIR, Waltham, MA, USA), and X-ray photoelectron spectroscopy (XPS, ESCALAB 250XI, Thermo Fisher Scientific, Waltham, MA, USA), respectively.

## 4. Conclusions

In summary, various nanocomposites based on different 2D materials (Ti_3_C_2_, Ti_3_AlC_2_, WS_2_, and MoSe_2_) and spherical NiCo_2_O_4_ were synthesized using the hydrothermal method. Different characterization strategies such as XRD, SEM, EDS, TEM, HRTEM, BET, FTIR, and XPS were used to examine the synthesized materials’ crystal structures, morphologies, and chemical states. The gas sensing properties were investigated thoroughly, four sensor sheets were fabricated from the synthesized samples, and the results showed that the MXene Ti_3_C_2_T_x_/NiCo_2_O_4_-based gas sensor detected the highest response (16) toward 10 ppm H_2_S at room temperature. In comparison, the sensor detected the highest response (9.8) toward 4000 ppm butane at 90 °C compared with the other three fabricated sensors (Ti_3_AlC_2_, WS_2_, and MoSe_2_/NiCo_2_O_4_). The Ti_3_C_2_T_x_/NiCo_2_O_4_-based gas sensor also showed minimum detection limits of 0.1 ppm H_2_S at room temperature and 5 ppm butane at 90 °C; furthermore, the sensor generated sensational selectivity and great stability/reproducibility. Moreover, the sensor detected the highest response toward 10 ppm H_2_S when the relative humidity was 25%, and the response was decreased by increasing the RH. The gas-sensing detection at low temperatures with the current sensor could be a promising candidate for H_2_S and butane detection because the sensor showed an almost linear relationship between H_2_S/butane concentrations and its response.

## Data Availability

The data will be made available upon reasonable request.
